# Hepatosplenic gamma delta T-cell lymphoma in a boy with visceral leishmaniasis: a case report

**DOI:** 10.1186/1752-1947-7-269

**Published:** 2013-12-13

**Authors:** Deepti Mutreja, Mrinalini Kotru, Mukul Aggarwal, Narender Tejwani, Rahul Kumar Sharma, Hara Prasad Pati

**Affiliations:** 1 Department of Hematology, All India Institute of Medical Sciences, New Delhi, India

**Keywords:** Hepatosplenic T-cell lymphoma, Immunosuppression, Visceral leishmaniasis

## Abstract

**Introduction:**

Hepatosplenic gamma delta T-cell lymphoma is a rare peripheral T-cell lymphoma of cytotoxic T-cell origin with an aggressive clinical course. Chronic immunosuppression has been proposed as a possible pathogenetic mechanism. No association of hepatosplenic gamma delta T-cell lymphoma with visceral leishmaniasis has been described in the past. We describe a case of an adolescent boy with hepatosplenic gamma delta T-cell lymphoma with leukemic presentation, who was diagnosed to have visceral leishmaniasis, 9 months prior to presentation at our center. To the best of our knowledge this is the first report of hepatosplenic gamma delta T-cell lymphoma with a prior history of visceral leishmaniasis in the medical literature.

**Case presentation:**

A 13-year-old Indian boy presented to the hematology out-patient department with a history of progressive abdominal distension of 9 months’ duration and low grade fever of 2 months’ duration. He was a known case of visceral leishmaniasis and was treated with some clinical improvement in the past. However, his symptoms recurred and he was diagnosed to have hepatosplenic gamma delta T-cell lymphoma at our center. Cytogenetic analysis showed characteristic karyotype of isochromosome 7.

**Conclusions:**

Chronic antigen stimulation due to visceral leishmaniasis may have led to an expansion of gamma delta T cells in our patient, and immunophenotypic analysis of bone marrow aspirate and characteristic karyotype helped to achieve the diagnosis. The aim of this case report is to highlight the rare association of hepatosplenic T-cell lymphoma with visceral leishmaniasis.

## Introduction

Gamma delta (γδ) T cells constitute 1% to 5% of the circulating lymphocytes in human blood [1]. These cells preferentially home in on some epithelial rich tissues and sinusoidal areas of the splenic red pulp where they represent up to 30% of the whole T-cell population. Hepatosplenic γδ T-cell lymphoma (HSTCL) is a rare aggressive subtype of extranodal lymphoma, representing less than 5% of all peripheral T-cell lymphomas. It often affects young males with a median age of onset of approximately 35 years [1,2]. Approximately 150 cases have been described in the literature since the initial description of this disease in 1990 [3]. The etiology of the disease is unknown, however, association with chronic immunosuppression including solid organ transplantation, hematopoietic neoplasms, inflammatory bowel disease, and malaria infection has been described [4]. Lymphomas of B-cell lineage have earlier been described in association with visceral leishmaniasis (VL) [5,6]. Also association of VL with hepatosplenic T-cell lymphoma has been reported in a canine in the past [7]. However, there has been no description of any T-cell lymphoma associated with VL in humans. We describe the rare association of HSTCL in a young adolescent male with a prior history of VL, the first report of this kind to date.

## Case presentation

A 13-year-old Indian boy, resident of Bihar, India, presented to the hematology out-patient department with a history of progressive abdominal distension of 9 months’ duration and low grade fever of 2 months’ duration. He complained of associated weakness, fatigue and weight loss. There was no history of jaundice or enlarged lymph nodes. Evaluation at another center at presentation had shown amastigote forms of *Leishmania* in his bone marrow (BM) for which he was treated with liposomal amphotericin B 0.6mg/kg for a total of 21 days with some clinical improvement. At 8 months after the end of treatment, he presented with recurrence of fever and anemia. A repeat aspiration done at this stage showed no Leishman-Donovan (LD) bodies, however, 30% blast-like cells were encountered. With a clinical suspicion of hematolymphoid malignancy, he was referred to our center.

Examination at our center revealed a thin boy with pallor and petechial rash over his chest. No enlarged lymph nodes were palpated. An abdominal examination revealed distended abdomen with multiple dilated veins. His liver was enlarged 10cm and his spleen was enlarged 20cm below the costal margin extending up to his right iliac fossa. A computed tomography scan of his abdomen showed massive hepatosplenomegaly with prominence of portal vein and mild ascites. No enlarged mediastinal or retroperitoneal lymph nodes were identified.

**Figure 1:**
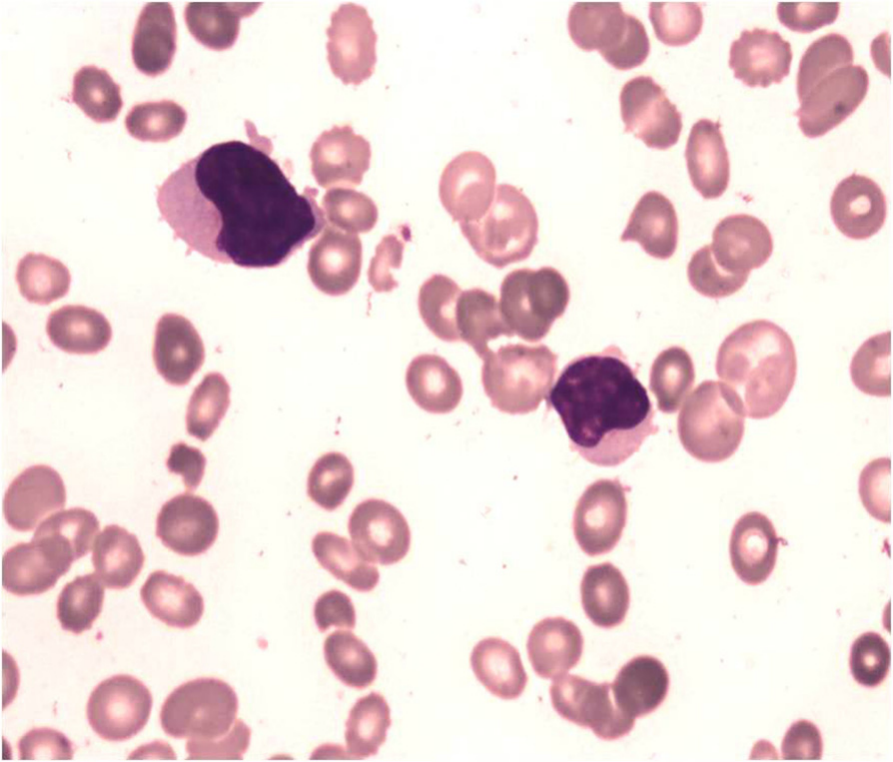
**Peripheral blood smear showing large atypical lymphoid cells with indented nuclei.** Jenner Giemsa, ×400.

**Figure 2:**
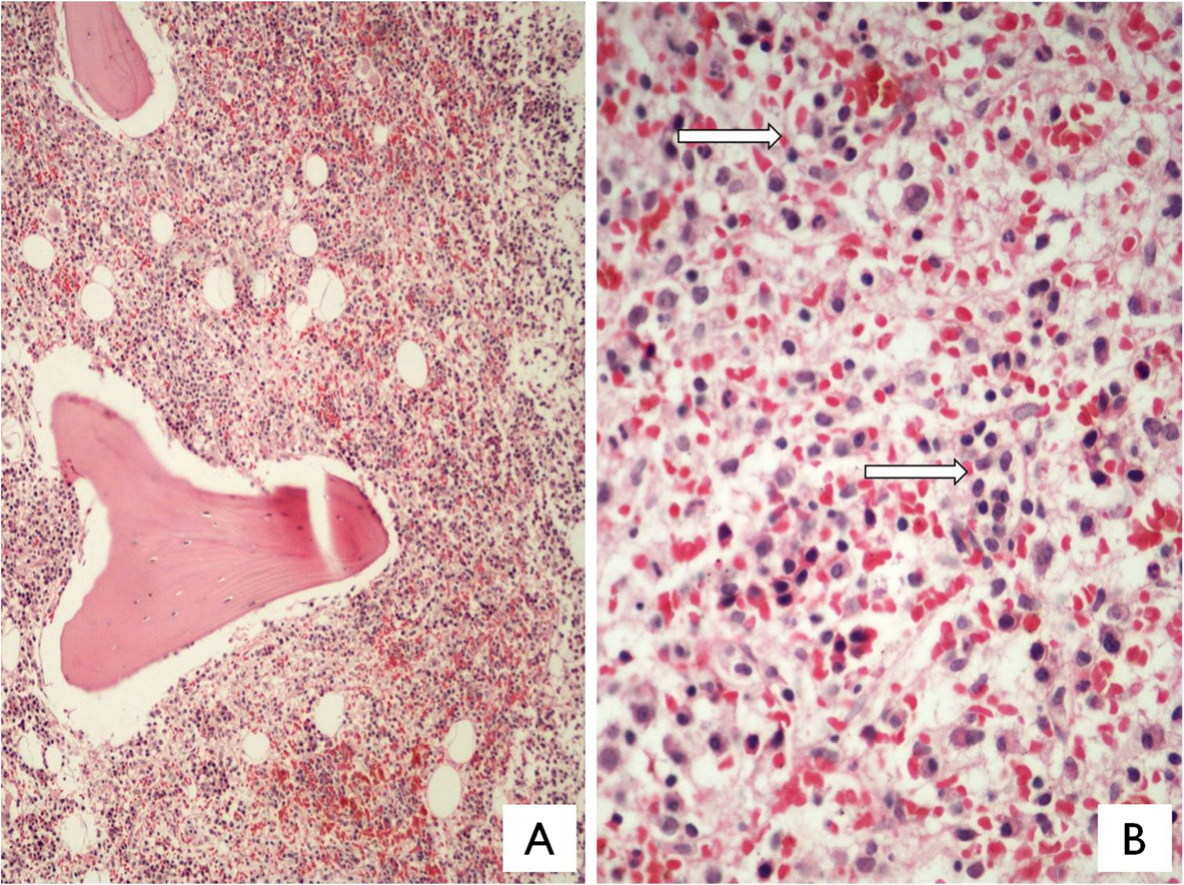
A: Bone marrow biopsy showing cellular marrow with diffuse intrasinusoidal and interstitial lymphoid cell infiltrates (Jenner Giemsa, ×100); B: Arrows indicating intrasinusoidal lymphoid cell infiltrate (Jenner Giemsa, ×400).

Investigations revealed hemoglobin of 76g/L, white blood cell count of 17.28×10^9^/L with 76% atypical medium to large-sized lymphocytes seen on peripheral blood smears (Figure 1), a platelet count of 40×10^9^/L, and elevated lactate dehydrogenase of 1024IU/L. Viral serology for hepatitis B, C and human immunodeficiency virus 1 and 2 was normal. A direct Coombs test was negative. A BM examination repeated at our center showed normoblastic erythroid precursors, normal myelogram and inadequate megakaryocytes. A predominant population (50%) of atypical large-sized lymphoid cells with oval to indented nuclei was seen. No LD bodies were demonstrated. Immunophenotyping of gated lymphoid cells on marrow cell suspension showed CD3+, CD2+, CD7+, CD56+, T-cell receptor (TCR) γδ+, TCR αβ–, and CD4– CD8–. Negativity for B cell markers (CD19, CD10, CD22, CD79a); myelomonocytic markers (myeloperoxidase, CD64, CD117) and immaturity markers (CD34, human leukocyte antigen-DR, terminal deoxynucleotidyl transferase) were seen. A BM biopsy showed monomorphic intrasinusoidal and interstitial infiltration of neoplastic lymphocytes (Figure 2A, 2B), which were highlighted by T-lineage markers on immunohistochemistry. Previous BM smears were reviewed and showed no atypical lymphoid cells. In view of the immunophenotype, a diagnosis of HSTCL in a background of VL was established. Cytogenetic evaluation on BM sample showed 45, X,–Y,i(7)(q10). The patient, however, had an episode of massive upper gastrointestinal bleeding and died prior to being started on chemotherapy. An autopsy was not performed.

## Discussion

HSTCL is characterized by significant hepatosplenomegaly, without lymphadenopathy. BM examination combined with immunophenotyping is sufficient for diagnosis, and splenectomy is unwarranted [2]. Chronic immunosuppression has been proposed as a possible pathogenetic mechanism in HSTCL [2,5].

An association between hematolymphoid malignancies and VL has been described rarely [5,6]. Leishmaniasis is caused by flagellated protozoa of the genus *Leishmania* and is transmitted to humans by the bite of a sand fly vector. VL is a systemic disease with gradual onset of fever, pancytopenia, hepatosplenomegaly, and weight loss. The incubation period can range from weeks to a year. VL is fatal if left untreated [6]. Here, we report a case of HSTCL that developed several months after VL infection. Bihar, our patient’s native state, is an endemic area for VL leishmaniasis in India. This raises the suspicion of a possible correlation between HSTCL and VL, and may represent different stages of clonal selection and propagation, in the context of a chronic lymphoproliferative process. The recognition of LD bodies in the BM several months before the immunophenotypically confirmed diagnosis of HSTCL is more likely to reflect chronic VL than a pre-existing HSTCL, which was unrecognized and untreated in the past.

Other infections that have been described associated with HSTCL are falciparum malaria and Epstein–Barr virus infections [2]. Of interest, both of these infections and VL have been associated with the expansion of T cells, presumably as a result of chronic antigenic stimulation. It is possible that γδ T cells are involved in the host response to *Leishmania* parasite, and prolonged antigenic stimulation and chronic immunosuppression, typical of VL, may play a role in the pathogenesis of HSTCL. In view of the characteristic cytogenetic abnormality of isochromosome 7, there still remains a possibility that the association of VL with HSTCL may be a chance occurrence. Nevertheless, the association with VL cannot be ignored and has been described to occur in a dog in the past [7].

Our patient had a leukemic presentation with 70% blast-like cells in the peripheral blood. This is more frequently seen during the late stages of this disease or after splenectomy. BM infiltration usually in HSTCL is also seen in the later stages of the disease, and the need for repeated BM biopsy has been indicated [8]. Different patterns of involvement have been reported, including exclusively sinusoidal, interstitial, and mixed sinusoidal and interstitial [9]. Immunophenotypic analysis of BM helps to clinch the diagnosis because it is helpful for demonstration of aberrant T-cell lineage. In our patient, the initial BM and biopsy specimen were negative morphologically and immunophenotypic analysis on BM was not performed.

## Conclusion

In conclusion, chronic antigen stimulation due to VL may have led to the expansion of γδ T cells in our patient and immunophenotypic analysis of BM and characteristic karyotype helped to achieve the diagnosis of HSTCL.

## Consent

Written informed consent was obtained from the deceased patient’s next of kin for publication of this case report and accompanying images. A copy of the written consent is available for review by the Editor-in-Chief of this journal.

## Abbreviations

γδ: Gamma delta; BM: Bone marrow; HSTCL: Hepatosplenic γδ T-cell lymphoma; LD: Leishman-Donovan; VL: Visceral leishmaniasis.

## Competing interests

The authors declare that they have no competing interests.

## Authors’ contributions

All authors analyzed and interpreted the patient data regarding the hematological disease. DM and MK performed the immunophenotypic analysis and were major contributors in writing the manuscript. MA and NT were involved in the clinical evaluation and histological examination of the bone marrow respectively. All authors read and approved the final manuscript.
